# Equilibrium and thermodynamic investigation of biosorption of nickel from water by activated carbon made from palm kernel chaff

**DOI:** 10.1038/s41598-021-86932-6

**Published:** 2021-04-08

**Authors:** Chidozie Charles Nnaji, Akambende Edward Agim, Cordelia Nnennaya Mama, PraiseGod Chidozie Emenike, Nkpa Mba Ogarekpe

**Affiliations:** 1 Department of Civil Engineering, University of Nigeria, Nsukka, Nigeria; 2 Faculty of Engineering and Built Environment, University of Johannesburg, Johannesburg, South Africa; 3 Department of Computing and Engineering, University of Huddersfield, Queensgate, Huddersfield, HD1 3DH UK; 4 Department of Civil Engineering, Covenant University, Ota, Ogun State Nigeria; 5 Cranfield Water Science Institute, School of Water, Energy, and Environment, Cranfield University, Bedford, MK43 0AL UK; 6 Department of Civil Engineering, Cross River University of Technology, Calabar, Nigeria

**Keywords:** Environmental sciences, Chemistry, Engineering

## Abstract

Novel biosorbents were derived from a waste product of palm kernel oil extraction known as palm kernel chaff (PKC). One portion of the PKC was carbonized in a furnace and then activated chemically, while the other half was activated without carbonization. Both were designated as CPKC and UPKC, respectively. The two biosorbents so produced were then used to conduct batch equilibrium and kinetic sorption studies at 30 °C, 35 °C and 40 °C and pH 3.0 and 9.0 for an agitation period of 5, 10, 20, 40, 60, 90, and 120 min. The Koble-Corrigan, Dubinin-Radushkevich, and the Freundlich isotherms fitted the experimental data very well with R^2^ values of 0.97 to 1.0, 0.95 to 1.0, and 0.96 to 1.0, respectively. The linear type II Langmuir isotherm performed much better (0.96 ≤ R^2^ ≤ 1.0) than the nonlinear isotherm. The maximum sorption capacity was obtained as 120.6 mg/g using CPKC at pH 9.0 and 35 °C. The Langmuir separation coefficient values (0.022 ≤ R_L_ ≤ 0.926) show that the sorption of nickel to PKC is favorable. The most favorable sorption condition was found for CPKC at pH 9 and temperature of 40 °C. The values of sorption energy (8.21 ≤ E ≤ 14.27) and the isosteric heat of sorption (−133.09 ≤ ∆H_x_ ≤ −17.92) indicate that the mode of sorption is mostly ion exchange. Thermodynamic parameters also show that the process is exothermic and entropy-driven. The pseudo-second-order kinetic model shows the best correlation compared to the other kinetic models. The coefficient of correlation for the pseudo-second-order model was mostly within the range of 0.999–1.000 for 90% of all kinetic studies carried out.

## Introduction

Aggressive industrialization has given rise to the heightened concentration of heavy metals in both surface and ground waters. The presence of heavy metals in the human environment in concentrations above permissible limits constitutes severe environmental and public health risk and can cause irreversible damage to plant and animal health^1^. Nickel is a naturally occurring heavy metal existing in various mineral forms and may be found throughout the environment, including rivers, lakes, oceans, soil, air, drinking water, plants, and animals. Soil and sediment are the primary receptacles for nickel, but mobilization may occur depending on physicochemical characteristics of the soil^2–5^. Nickel is used extensively in the electronic and metallurgical industries, specifically in electroplating, nickel–cadmium batteries, printed circuit boards, liquid crystal displays; and is emitted into the environment through anthropogenic sources such as fuel combustion, smelting, sewage, and solid waste management and fertilizer application^1,6–9^. Effluent from such industrial processes requires treatment to permissible levels before discharge. The removal of metals from effluents is a major problem due to the difficulty in treatment by conventional treatment methods. Some evidence suggests that nickel may be an essential trace element for mammals^10^, though its concentration in water above permissible limits is of concern to public health. As for most metals, the hazardous nature of nickel hinges on its solubility and exposure route^11^. The inhalation route to some nickel compounds often results in toxic effects in the respiratory region and impacts the immune system^3,10,12–14^. When consumed in massive amounts (> 0.5 g) through oral intake, some forms of nickel pose acute toxicity to humans, resulting in elevated skin irritation, cardiovascular diseases, as well as cancer^15–18^. Several treatment processes such as chemical precipitation, adsorption, ion exchange, and membrane filtration have been deployed over the years to eliminate metal(oid)s in water and industrial wastewaters. However, most of these techniques have some disadvantages, such as complicated treatment processes, high cost, and high energy requirement^19,20^.

Over the years, biosorption has become a lucrative and promising wastewater treatment technique for extracting metal(oid)s. According to Saha and Chowdhury^21^, the effectiveness of the adsorption technique has been outstanding in the removal of soluble heavy metal ions, synthetic dye molecules, and other toxic chemicals from aqueous solution. Owing to the fact that adsorption is a cost-effective, highly efficient, and easily implemented method, its implementation made it a welcomed alternative to conventional treatment processes^22^. Activated carbon, being one of the popularly used adsorbents, is mainly composed of carbonaceous materials with improved porosity to adsorb chemical ions. Its internal surface area and relatively high mechanical strength make it suitable for removing heavy metals from wastewaters^20,23–25^. As a result of the advancement in adsorption technology, the demand for activated carbon is skyrocketing, and over time, making it an expensive material. A technology showing good potential applicability is converting nanoparticles to hybrid hydrogels to remove various aquatic pollutants. Hydrogel is considered a 3-dimensional polymer synthesized through the reaction of one or more monomers. Its 3-D network and porous attributes reflect on the hydrophilic property and adsorptive capacity to extract a commeasurable quantity of biological fluids^26,27^. However, low-cost forest and agricultural wastes without or with little processing are considered promising biosorbents for heavy metals due to their high surface areas, microporous attributes, and surface chemical nature^28–30^. The need to explore the possibility of waste-derived biosorbent is more pressing now than ever in the light of the environmental consequences of felling trees to make activated carbon. The conversion of organic waste materials into activated carbon will also reduce the ever-increasing burden of solid waste management. Agricultural wastes and other organic origin waste have been found suitable for heavy metals removal from water by adsorption. It is believed that efficient conversion and utilization of waste materials will go a long way to mitigate environmental pollution and degradation as well as reducing the cost of waste treatment. Biosorbents like coconut shell, nutshells, oil palm waste, pine needles, sawdust, waste straw, rice husk, peanut hulls, hazelnut shells, almond shells, peach stones, tea dust leaves, apple wastes, sugarcane bagasse, coffee grounds, banana and orange peels, sugar beet pulp and different other materials have all been used as biosorbents with varying degrees of success^19,20,23,25,28,31–35^.

**Figure 1:**
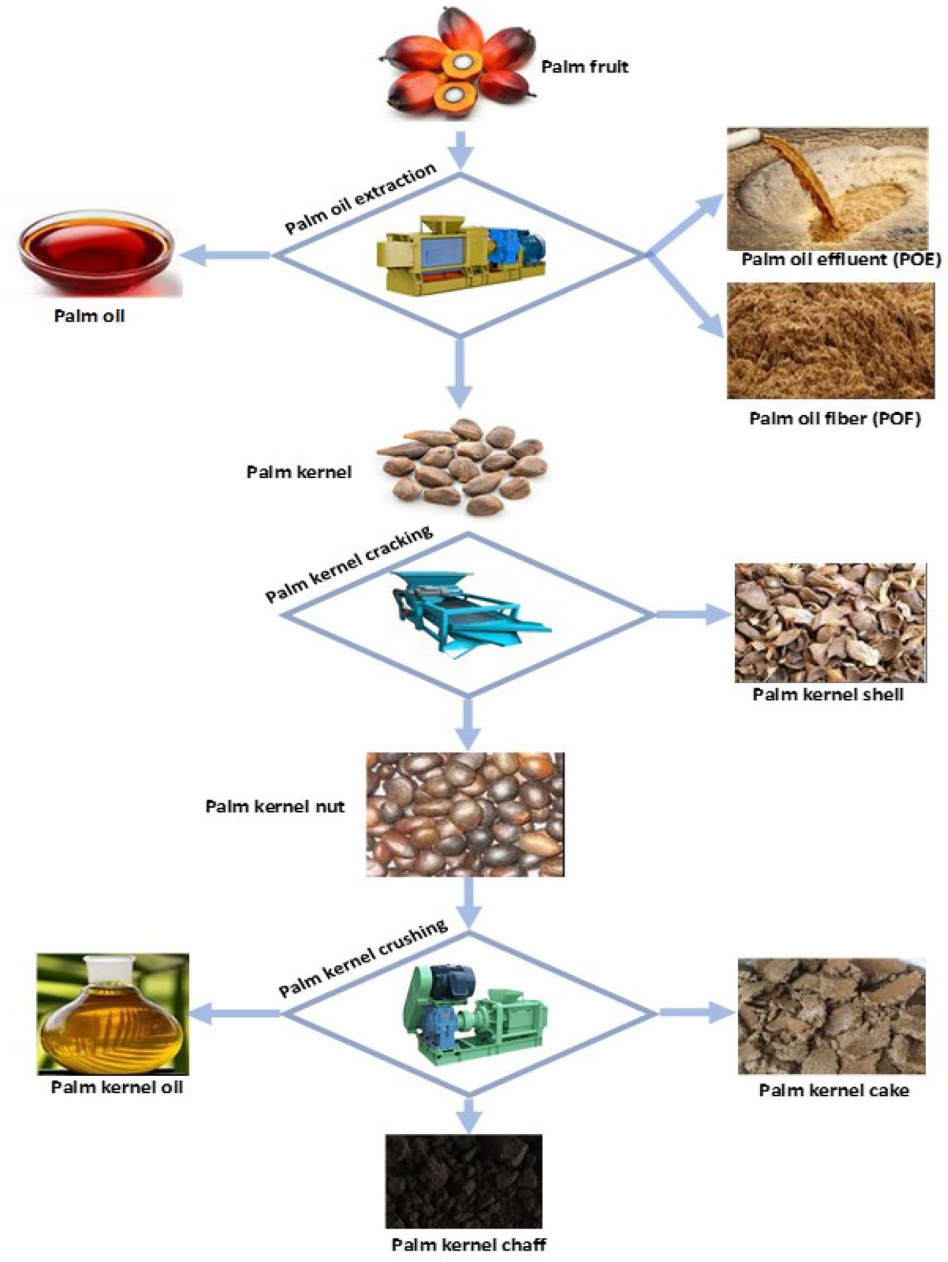
Process flow for primary components of palm fruit.

This study focuses on the beneficial application of palm kernel chaff, a by-product of palm kernel oil extraction. During extraction of palm kernel oil, palm cake and palm oil chaff are generated as by-products. While palm kernel cake is usually the by-product of palm kernel oil extraction from screw press, palm kernel chaff usually results from local extraction (Fig. 1). The local process is two-staged. The first stage consists of heating the palm kernel to expel loosely bound oil, while the second stage involves crushing the charred palm kernel in a screw press. The resulting slurry is then separated into oil and chaff. Palm kernel cake is used either as high-protein feed for dairy cattle or burned in boilers to generate electricity for palm oil mills and surrounding villages. Though the palm kernel cake is partly composed of chaff, we have thought it necessary to distinguish between the two because palm kernel cake is more valuable than palm kernel chaff. Palm kernel cake is a source of fiber, protein, and other essential elements such as magnesium, iron, calcium, and zinc, making it well-suited for animal feed^36^. Studies have been conducted using palm kernel shell, oil palm fibre, and palm kernel cake as adsorbent^37–39^. None was found on the use of palm kernel chaff as an adsorbent. This study undertook a comprehensive investigation of the physicochemical conditions for biosorption of nickel from water by activated carbon made from palm kernel chaff (PKC), a waste biosorbent.

## Methodology

### Collection and preparation of adsorbents

Palm kernel chaff (PKC) was obtained from a local palm kernel extraction point in Nsukka, Enugu State, Nigeria. The PKC was air-dried for five days and then sieved using standard sieve numbers 8, 10. 16, 20, 22, 25 and 30 corresponding to 2.36 mm, 2.0 mm, 1.18 mm, 0.85 mm, 0.71 mm, 0.6 mm and 0.36 mm respectively. Particles passing through sieve size 0.36 mm were collected and used for the experiment. The sample was washed severally with de-ionized water and dried in a vacuum oven at 103 °C until it was scorched. The sample was then divided into two equal parts: A and B. This was done in order to ascertain the effect of carbonization on the sorption characteristics of PKC. Sample A was further carbonized in a furnace at a controlled temperature of 250 °C for 2 h and then washed with de-ionized water. It was then dried in a vacuum oven until completely dry. Sample B was not carbonized in a furnace. Samples A and B were separately activated chemically by soaking and slurry for 3 h using 0.5 M hydrochloric acid. The gases formed due to exothermic reaction were allowed to escape for 10 min, and the mixture was covered with a lid for 3 h. This was followed by repeated washing with de-ionized water for complete removal of traces of the chemical and oven drying for 3 h at 110 °C. The samples were then milled separately in a porcelain jar to increase the specific surface area by reducing lumps. After that, the samples were then cooled and placed in two separate clearly labelled air-tight plastic containers to keep away moisture and dust for further use.

Sample A was designated as carbonized palm kernel chaff (CPKC), while sample B was designated as uncarbonized palm kernel chaff (UPKC). Ten grams (10 g) of samples A and B were extracted and sent to the National Geosciences Research Laboratory Kaduna, Nigeria, for chemical composition analysis by x-ray fluorescence (XRF). The point of zero charge (pH_zpc_) of the adsorbents was determined in the Public Health Laboratory of the Department of Civil Engineering, University of Nigeria. The point of zero charge was used to investigate the variation of the surface charge of palm kernel chaff with a change in the solution's pH. Following the method described by Singh et al.^40^, NaCl (0.01 M) was prepared, and 50 ml was dispensed into six 250 ml flasks. The solution's pH was adjusted using NaOH and HCl to a range of 2 to 12, and 0.2 g of the adsorbent was added to the solution and allowed to stand for 48 h. The pH of each solution was then determined using a portable pH meter, and this final pH was recorded. A plot of the initial pH versus the final pH was prepared, and the point of intersection between this line and the 45° line passing through the origin gave the value of the point of zero charge. This was done for both the carbonized palm kernel chaff and the uncarbonized palm kernel chaff.

### Preparation of adsorbate and batch equilibrium experiment

Analytical grade stock solution of nickel nitrate was utilized, and six beakers of stock nickel solutions with concentrations of 10, 30, 50, 70, 100, and 120 mg/L were prepared using appropriate amounts of the stock solution. The pH was adjusted to 3.0 using 0.5 M HCl and a pH meter. The completely mixed batch reactor (CMBR) technique was used to investigate the adsorption of Nickel from water by putting 100 mg of adsorbent in 100 ml nickel solution. The batch adsorption was conducted at temperatures of 30 °C, 35 °C, and 40 °C for both CPKC and UPKC for an agitation period of 5, 10, 20, 40, 60, 90, and 120 min. The same procedure was repeated for the same concentrations of nickel solution adjusted to pH 9 using 0.5 M NaOH buffer solutions. At equilibrium, the adsorbent was separated from the solution by filtration with Whatman filter paper number (0.45 µm). The equilibrium concentration of nickel ion was determined using an Atomic Absorption Spectroscopy Machine (Shimadzu, AA 7000). All equipment and their accessories, as well as reagents (except the stock nickel nitrate solution) used, were made available by the National Center for Energy Research and Development, University of Nigeria, Nsukka, Nigeria.

### Analyses of experimental results

At the end of the equilibrium experiments, the quantity of Ni (II) adsorbed was determined. The amount of metal adsorbed per gram of adsorbent at equilibrium was calculated as follows.

1 $${q}_{e}= \frac{{C}_{o}- {C}_{e}}{m} V$$

where *q*_*e*_ is the quantity of nickel removed per unit mass of adsorbent (mg/g), *m* is the mass of adsorbent (g), *V* is the volume of adsorbate (*L*), *C*_*o*_ is the initial concentration of adsorbate (mg/l), and *C*_*e*_ is the equilibrium concentration of adsorbate in solution. The experimental data were fitted to six isotherms, namely: Langmuir, Freundlich, Temkin and Dubinin Radushkevich (D-R), Florry-Huggins, and Koble-Corrigan isotherms. Adsorption isotherms provide insight into the adsorbent's sorption mechanisms, surface properties, and affinity for the adsorbent^41^. Langmuir isotherm represents the equilibrium distribution of metal ions between the solid and liquid phases by the formation of a monolayer on the adsorbent surface containing a finite number of identical sites with uniform energies of adsorption^42^. The expression for the Langmuir isotherm is given as Eq. 2, where *C*_*e*_ is the equilibrium concentration of solute (mg/l), *q*_*e*_ is the quantity of solute in the solid phase at equilibrium (mg/g), ${q}_{max}$ is the maximum adsorption capacity (mg/g) and ${K}_{L}$ is the Langmuir constant. Both the non-linear and the linear forms of Langmuir isotherm were considered. In this study, the Langmuir types I, II, III and IV were used to fit the experimental data and are given as Eqs. (3) to (6).

Langmuir Isotherm

2 $${q}_{e}= \frac{{q}_{max}{K}_{L}{C}_{e}}{1+{K}_{L}{C}_{e}}$$

Langmuir Type I

3 $$\frac{{C}_{e}}{{q}_{e}}=\frac{{C}_{e}}{{q}_{max}}+\frac{1}{{q}_{max}{K}_{L}}$$

Langmuir Type II–Lineweaver-Burke

4 $$\frac{1}{{q}_{e}}=\frac{{C}_{e}}{{q}_{max}{K}_{L}}+\frac{1}{{q}_{max}}$$

Langmuir Type II–Eadie-Hoffsie

5 $${q}_{e}={q}_{max}-\frac{{q}_{e}}{{K}_{L}{C}_{e}}$$

Langmuir Type IV–Scatchard

6 $$\frac{{q}_{e}}{{C}_{e}}={q}_{max}{K}_{L}-{K}_{L}{q}_{e}$$

The Freundlich adsorption isotherm describes the adsorption characteristics of a heterogeneous surface. This isotherm recognizes the possibility of surface heterogeneity and intermolecular interactions between adsorbed molecules^43^. Freundlich and Langmuir's isotherms have been the most widely used isotherms used by researchers to investigate the performance of adsorbents. The nonlinear and linear forms of Freundlich isotherm are given as Eqs. (7) and (8), respectively, where $n$ is the Freundlich intensity parameter and ${K}_{F}$ = Freundlich constant which is indicative of the relative adsorption capacity of the adsorbent (mg/g)/(mg/L)^*n*^.

7 $${q}_{e}={K}_{F}{{C}_{e}}^{1/n}$$

8 $$Ln{q}_{e}=Ln{K}_{F}+\frac{1}{n}Ln{C}_{e}$$

The Temkin isotherm contains a factor that explicitly takes into account adsorbent–adsorbate interactions. By ignoring the extremely low and large value of concentrations, the model assumes that the heat of adsorption (a function of temperature) of all molecules in the layer would decrease linearly rather than logarithmically with coverage^44^. The nonlinear and linear forms of Temkin isotherm are given as Eqs. (9) and (10), where $B$ is the Temkin constant while *A* is the equilibrium binding constant (L/g).

9 $${q}_{e}=B\mathrm{ln}\left(A{C}_{e}\right)$$

10 $${q}_{e}=BLnA+BLn{C}_{e}$$

The Dubinin–Radushkevich (D-R) isotherm expresses the adsorption mechanism with a Gaussian energy distribution onto a heterogeneous surface. The model has often successfully fitted high solute activities and the intermediate range of concentration data well^44^. The D-R isotherm is generally applied to express the adsorption process occurring onto both homogenous and heterogeneous surfaces^45^. It is a semi-empirical isotherm that assumes that sorption follows a pore-filling mechanism^46^. The use of the D-R isotherm is also essential for investigating the nature of sorption using the sorption energy values (*E*). The expression for the D-R isotherm is given as Eq. (10), where ${q}_{m}$ is the maximum sorption capacity (mg/l), $\beta$ is the activity coefficient related to sorption energy, R (J/molK) is indicative of the gas constant, T is the Kelvin temperature and $\varepsilon$ is the Polanyi potential in mol^2^/J^2^. The linearized form of the D-R isotherm is presented in Eq. (12).

11 $${q}_{e}={q}_{m}exp\left(-\beta {\varepsilon }^{2}\right)$$

12 $$\varepsilon =RT\mathrm{ln}\left(1+\frac{1}{{C}_{e}}\right)$$

13 $$Ln{q}_{e}=Ln{q}_{m}-\beta {\varepsilon }^{2}$$

Flory–Huggins isotherm describes the degree of surface coverage characteristics of the adsorbate on the adsorbent^46,47^. Theta ($\theta$) is the degree of surface coverage, $n$ is the Flory–Huggins exponent and $K$ is the equilibrium constant (L/mg). The Flory-Hugging isotherm is presented as Eq. (13) which was then log-transformed to obtain the linear form given in Eq. (14).

14 $$\frac{\theta }{{C}_{0}}=K{\left(1-\theta \right)}^{n}$$

15 $$Ln\left(\frac{\theta }{{C}_{0}}\right)=\mathrm{ln}K+n\mathrm{ln}\left(1-\theta \right)$$

The degree of surface coverage used for analysis is expressed as

16 $$\theta =1-\frac{{C}_{e}}{{C}_{0}}$$

Koble-Corrigan isotherm is a three-parameter isotherm resulting from the combination of the Langmuir and Freundlich isotherms.

17 $${q}_{e}=\frac{a{C}_{e}^{n}}{1+b{C}_{e}^{n}}$$

## Results and discussion

### Chemical composition of PKC on the adsorption of Ni

Palm kernel chaff (PKC) is an abundant agricultural by-product that consists of 20–30% cellulose^48^. CPKC and UPKC are black and dark brown, respectively. XRF results show that the dominant elements in UPKC are S (83.86%), Ti (10.08%), and Fe (3.96), while those in CPKC are Ca (47.16%), K (23.82%), S (15.69), Fe (5.10%), Ti (4.82%) and Mn (1.16%). It can be seen that carbonization drastically reduced the Sulphur content of PKC. The results show that the dominant elements are Sulphur, calcium, titanium, potassium, and iron. Sulphur-containing compounds ionize to produce sulphites, sulphates, and sulphides (with 2-anions), constituting counterions to Nickel (Ni^2+^). The counterions from the PKC are adsorbed onto the surface of the Ni^2+^. This, therefore, results in the neutralization of the repulsive charges on the ionized Nickel atoms. Calcium, iron, and the other elements react with the alkalinity in water to form insoluble and slightly soluble hydroxides precipitated out of the solution. Adsorption can occur effectively through chemical bonds (covalent or ionic) between the surface hydroxyl group and the adsorbate ions (cations or anions)^49^.

The neutralized nickel and the precipitates are then duly adsorbed at the adsorption sites. The chemical composition of the PKC coupled with the impact of high alkalinity on chemical precipitation further corroborates the strong affinity and favorable sorption of Ni (II) onto PKC at pH 9.

### FT-IR of PKC samples

**Figure 2:**
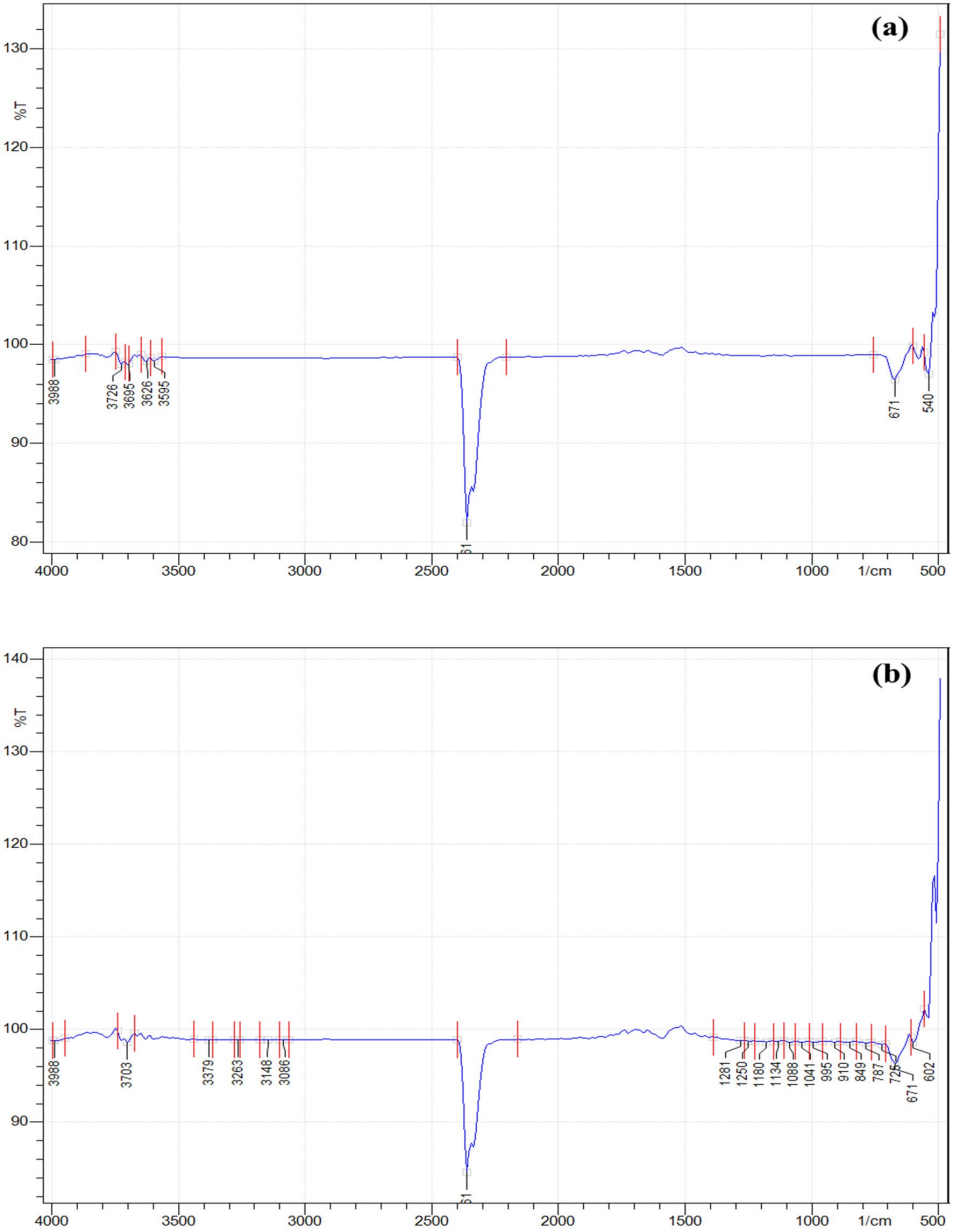
FT-IR representation of IR signals at spectra of (**a**) UPKC surface and (**b**) CPKC Surface.

In relation to the varying characteristics of the signal bands identified at the surfaces of CPKC and UPKC, an IR band of 2361 cm^−1^ was identified in both states, which corresponds to the distending vibration of the –CH_2_ or –CH_3_ groups. At the CKPC surface, a notable band having a signal of 3703 cm^−1^ was identified as well, and at the UKPC surface, bands 3726 cm^−1^, 3695 cm^−1^, 3626 cm^−1^, and 3595 cm^−1^ was observed (see Fig. 2a and b). These bands indicate the dominating signal of the OH groups. To validate the bands produced, bending vibrations were observed at 1582 cm^−1^ and 1281 cm^−1^ in the CPKC sample. These bands confirm the presence of acidic oxygen in the functional groups; the C=O and C–O distending vibration of the –COOH group had signals around 1800 cm^−1^ and 1415 cm^−1^. The benefits of the acidic oxygen present in the functional groups are that they might be responsible for the adsorption of Nickel. It is worthy to note that the more substantial peaks corresponding to the –OH and –COOH groups explain the chemical modification of the samples could enhance the formation of a functional group that is characterized by high acidic oxygen content. The literature has also been established that chemical adsorption is propagated by surface complexation and ion exchange mechanism^50^. However, this can be possible when the surface of the adsorbent is endowed with hydroxyl and carboxyl functional groups. Thus, the presence of these functional groups (–OH and –COOH) at the surface of CKPC is liable to promote nickel adsorption.

### Efficiency of sorption of Ni (II) by PKC under various physicochemical conditions

Contact time had a considerable effect on Ni (II) biosorption by chemically activated CPKC and UPKC. An increase in contact time resulted in a corresponding increase in biosorption efficiency up to the equilibrium time. Generally, the sorption rate was slow and gradual at pH 9 for both UPKC and CPKC, while at pH 3, the sorption efficiency was fairly rapid. The percentage removal of Ni (II) by CPKC at pH 9 and 40 °C was 100%, 97.2%, 95.9%, 92.8%, 89% and 84.9% at initial concentrations of 10 mg/l, 30 mg/l, 50 mg/l, 70 mg/l, 100 mg/l and 120 mg/l. These were higher than the values of 97.9%, 96.5%, 91.8%, 83.5%, 76.9%, and 71.5%, respectively, obtained for UPKC at the same pH and temperature. The corresponding percentage removal for CPKC at pH 3 was 66.9%, 58.1%, 55.6%, 50.2%, 47.4% and 44.6%, while the corresponding values for UPKC at pH 3 were 62.4%, 52.5%, 50.8%, 49.2%, 45.7% and 42.5%. In most cases, equilibrium was attained at 90 min and 120 min in very few cases. Smooth and continuous saturation of the adsorbent surface suggests a possible monolayer coverage of metal ions. This is attributed to the fixed number of active adsorption sites in any given adsorption system; hence, each active site can only adsorb one ion in a monolayer. This makes the intake of metal ions fairly rapid at the initial stage, slowing down as available active sites decrease. Chaouch et al.^51^ observed that the adsorption of metal ions from an aqueous compound is very much dependent on the pH. The pH governs important parameters such as the net surface charge on the adsorbent, the degree of ionization, the speciation of the adsorbent, predominant species, and the dissociation of active functional sites on the biosorbent^52^. In this study, high percentage adsorption of up to 100% was noted for CPKC within 60 min of contact at an initial concentration of 10 mg/l, the temperature of 40 °C, and pH 9. At pH 3, for the same adsorbent, metal ion concentration, and temperature, the percentage adsorption reduced to 66.92%, which amounts to a one-third reduction in efficiency. The lowest percentage sorption of 24.28% was recorded for UPKC at an initial nickel concentration of 120 mg/l, the temperature of 30 °C, and pH 3. The effect of pH on biosorption capacity in this study shows that at pH 9, the percentage of adsorption was better than at pH 3. The reduced sorption efficiency at pH 3 can be attributed to the predominance of hydrogen ions in the solution, which restricted the adsorption of metal ions. In addition, an increase in pH from 3 to 9 could result in the precipitation of Ni (II) ions due to hydroxide anions forming nickel hydroxide precipitate, thereby further reducing the concentration of Ni (II) ions in solution^53^. Arshadi et al.^54^ also noted that a low intake of heavy metals in an acidic medium might result from particle attrition, partial protonation of the functional groups, and the competition between hydrogen ions and metal ions for binding to the adsorption sites. The adsorption process is very dependent on pH and the concentration of the solution^43^. However, the effect of pH on adsorption varies according to the nature of the solute and the surface properties of the adsorbent. Hence, other researchers have obtained results that are either slightly different or even opposite to the results obtained in this study for different adsorbents and solutes. Raj^55^ observed that the adsorption of Ni (II) on activated carbon is efficient at pH less than 4.5 while Aikpokpodion et al.^56^ noted that the adsorption of Ni (II) on whole tea (*Camellia Simensis*) material recorded better efficiency at pH less than 5. Arshadi et al.^54^ recorded increase an adsorption efficiency of Cd (II), Cu (II) and Co (II) by barley stray ash when the pH of the solution was increased to 11. Generally, sorption efficiency seems to increase with increase in pH for adsorbents with point of zero charge less than the pH of the solution, while it will decrease for adsorbents with zero-point charge greater than the pH of the solution.

In this study, the removal efficiency of nickel was higher at low adsorbate concentrations, while the actual quantity of metal ion adsorbed increased when the adsorbate concentration increased. At pH 9, the sorption rate was very high in the first five minutes and then slowly attained equilibrium at 90 min. This behavior was pronounced for the lowest initial concentration of 10 mg/l at all experimental conditions. For instance, for CPKC at pH 9 and a temperature of 30 °C, sorption efficiency was 92.31% at 5 min and 97.85% at 90 min. Similarly, for the same physicochemical characteristics at pH 3, sorption efficiency was 14.1% at 5 min and 49.18% at 90 min. This behavior can be attributed to the high affinity between the positively charged Ni (II) ions and the negatively charged adsorbent surface at pH 9. Hence, Ni (II) and PKC's affinity were highest at the beginning of the adsorption process but decreased as the number of active sorption sites reduced.

### Adsorption isotherm

Adoption isotherms were used to analyze the experimental data with a view to gaining insight into the mechanism of adoption of Ni (II) onto PKC at various physicochemical conditions. Sorption isotherms are useful for evaluating the extent to which a sorption system can be improved and the optimization of operating conditions^57^. Both the linear and non-linear forms of the isotherms were employed to evaluate the adsorption process and performances (see Tables 1 and 2). Though the linear forms of the isotherm are easier to use, the process of linearization introduces a measure of error into the final result. The error distribution of linearized isotherms varies in accordance with the linearization techniques adopted^47^. He et al.^58^ observed that the transformation of non-linear isotherms to linear forms implicitly alters the error structure and may violate the error variance and normality assumptions of standard least squares. The effect of linearization has also been reported for Freundlich and Langmuir isotherms.

**Table 1:** Sorption isotherm parameters for non-linear fitting.

Model	Adsorbent	Carbonized PKC			Uncarbonized PKC			Carbonized PKC			Uncarbonized PKC		
	pH	9			9			3			3		
	Temp (°C)	30	35	40	30	35	40	30	35	40	30	35	40
Langmuir	*K*_*L*_ (L/mg)	0.197	0.347	0.369	0.058	0.097	0.316	0.011	0.017	0.016	0.015	0.008	0.012
	q_m_ (mg/g)	110.2	105.2	111.9	101.5	93.34	86.37	76.25	83.14	102.2	54.62	105.6	115.7
	R^2^	0.98	0.98	0.95	0.99	0.92	0.95	0.99	0.99	1.00	0.97	0.99	1.00
Koble-Corrigan	A	26.83	37.69	40.12	8.991	13.65	29.53	1.647	1.069	2.622	0.764	1.747	1.486
	n	0.54	0.73	0.45	0.73	0.27	0.46	0.71	1.10	0.80	1.01	0.71	0.96
	B	0.106	0.290	0.117	0.057	−0.203	0.151	0.003	0.015	0.014	0.014	−0.004	0.012
	R^2^	1.00	0.99	1.00	1.00	0.98	0.99	1.00	0.99	1.00	0.97	1.00	1.00
Freundlich	k (mg/g)/(mg/L)^*n*^	25.43	31.28	36.7	11.14	15.35	26.33	1.746	2.994	3.453	1.989	1.609	2.609
	n	2.39	2.66	2.79	1.98	2.26	2.97	1.46	1.56	1.52	1.63	1.35	1.41
	R^2^	1.00	0.97	1.00	0.99	0.98	0.99	1.00	0.98	1.00	0.96	1.00	0.99
Temkin	A (L/g)	5.21	5.03	5.34	1.07	2.77	7.64	0.23	0.28	0.34	0.25	0.22	0.28
	B	17.98	20.06	21.5	17.88	15.07	14.42	11.01	13.99	15.53	9.25	12.77	15.61
	R^2^	0.95	0.99	0.98	0.96	0.92	0.98	0.93	0.94	0.93	0.91	0.92	0.92
D-R	q_m_ (mg/l)	378.3	344.7	355.7	363.5	294.3	221.7	268.3	308.5	397	184.8	365.8	456.4
	β × 10^−3^	3.58	3.06	2.81	4.81	3.85	2.57	7.35	6.48	6.41	6.65	7.65	7.03
	R^2^	1.00	0.98	1.00	1.00	0.97	0.99	1.00	0.99	1.00	0.96	1.00	0.99
E (KJ/mol)		11.81	12.78	13.33	10.19	11.40	13.94	8.25	8.78	8.83	8.67	8.08	8.43
Florry-Huggins	K × 10^−5^ (L/mg)	52.11	67.48	175.5	33.45	23.63	23.1	9.016	8.594	13.62	29.76	1.392	11.92
	n	−1.36	−1.26	−0.75	−2.54	−2.01	−1.55	−9.31	−7.27	−5.60	−7.62	−11.80	−6.39
	R^2^	0.99	0.52	1.00	1.00	0.95	0.95	1.00	0.99	1.00	0.99	0.99	0.99

**Table 2:** Sorption isotherm parameters for linear fitting.

Model	Adsorbent	Carbonized PKC			Uncarbonized PKC			Carbonized PKC			Uncarbonized PKC		
	pH	9			9			3			3		
	Temp	30	35	40	30	35	40	30	35	40	30	35	40
Langmuir Type I	*K*_*L*_	0.251	0.327	0.563	0.071	0.131	0.300	0.014	0.017	0.021	0.018	0.012	0.015
	q_m_	106.3	108.1	107.4	95.4	88.2	90.3	64.3	80.9	89.6	48.8	82.8	99.8
	R^2^	0.98	0.99	0.97	0.98	0.94	0.98	0.95	0.95	0.96	0.92	0.93	0.93
Langmuir Type II	*K*_*L*_	0.73	0.30	0.77	0.13	0.33	0.80	0.02	0.03	0.03	0.03	0.02	0.03
	q_m_	71.2	120.6	90.2	71.8	65.4	68.7	48.9	62.2	65.9	36.0	63.8	65.2
	R^2^	0.99	0.99	0.96	0.99	0.99	0.99	1.00	0.99	1.00	0.99	1.00	0.99
Langmuir Type III	*K*_*L*_	0.542	0.420	0.645	0.099	0.285	0.675	0.018	0.021	0.027	0.024	0.014	0.021
	q_m_	83.7	99.1	97.3	82.3	71.0	75.1	56.3	71.8	77.6	41.5	71.6	80.8
	R^2^	0.80	0.90	0.84	0.90	0.81	0.87	0.90	0.85	0.89	0.78	0.88	0.82
Langmuir Type IV	*K*_*L*_	0.435	0.377	0.542	0.090	0.137	0.586	0.016	0.018	0.024	0.019	0.013	0.017
	q_m_	91.0	104.1	103.1	86.4	84.8	78.7	60.0	79.3	83.1	47.8	78.0	92.2
	R^2^	0.80	0.90	0.84	0.90	0.25	0.87	0.90	0.85	0.89	0.78	0.88	0.82
Generalized Isotherm	k	12.59	5.60	2.23	3.41	2.75	2.26	66.55	54.54	45.42	50.20	89.12	64.33
	n	0.90	0.82	1.11	0.84	1.02	0.95	0.91	0.97	0.92	0.92	0.91	0.92
	R^2^	0.99	0.93	0.80	0.97	0.98	0.94	1.00	0.99	1.00	0.99	0.93	0.80
Freundlich	k	22.51	24.75	36.02	9.04	14.79	22.69	1.59	2.20	3.34	1.66	1.53	2.21
	n	2.10	2.02	2.72	1.74	2.19	2.49	1.41	1.39	1.50	1.51	1.32	1.32
	R^2^	0.99	0.92	1.00	0.99	0.97	0.96	1.00	0.99	0.99	0.99	1.00	0.99
Temkin	a	5.21	5.03	5.34	1.07	2.77	7.64	0.23	0.28	0.25	0.25	0.22	0.22
	b	17.98	20.06	21.5	17.88	15.07	14.42	11.01	13.99	17.6	9.25	12.77	17.64
	R^2^	0.95	0.99	0.98	0.96	0.92	0.98	0.93	0.94	0.97	0.91	0.92	0.96
D-R	q_m_	403.8	523.7	348.3	398.6	276.4	209.1	243.5	340.0	409.5	178.8	314.2	522.2
	β × 10^−3^	3.71	3.83	2.78	5.04	3.70	2.46	7.06	6.77	6.49	6.57	7.20	7.41
	R^2^	1.00	0.95	1.00	1.00	0.98	0.99	1.00	0.99	1.00	0.99	1.00	1.00
	E (kJ/mol)	11.61	11.43	13.42	9.96	11.63	14.27	8.42	8.59	8.78	8.73	8.33	8.21
Florry-Huggins	K × 10^−5^	101.69	98.49	170.02	52.35	151.40	187.72	9.66	91.83	359.58	18.80	4.67	7.14
	n	−1.16	−1.09	−0.76	−2.30	−1.32	−0.95	−9.28	−9.18	−8.93	−8.44	−9.90	−6.99
	R^2^	0.99	0.80	1.00	1.00	0.95	0.96	1.00	0.95	0.99	0.97	0.98	0.98

**Figure 3:**
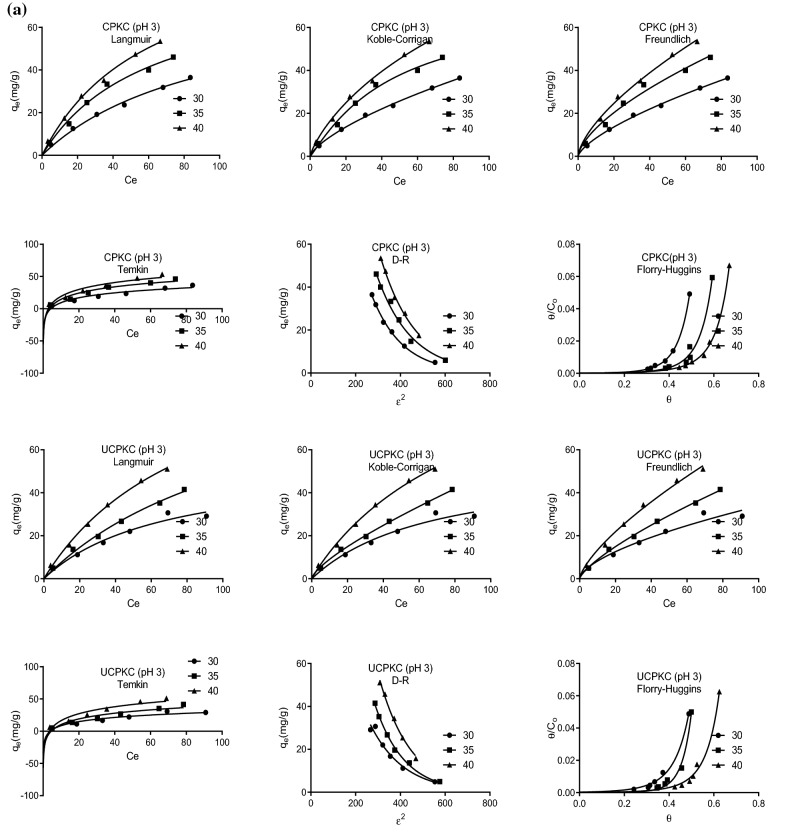

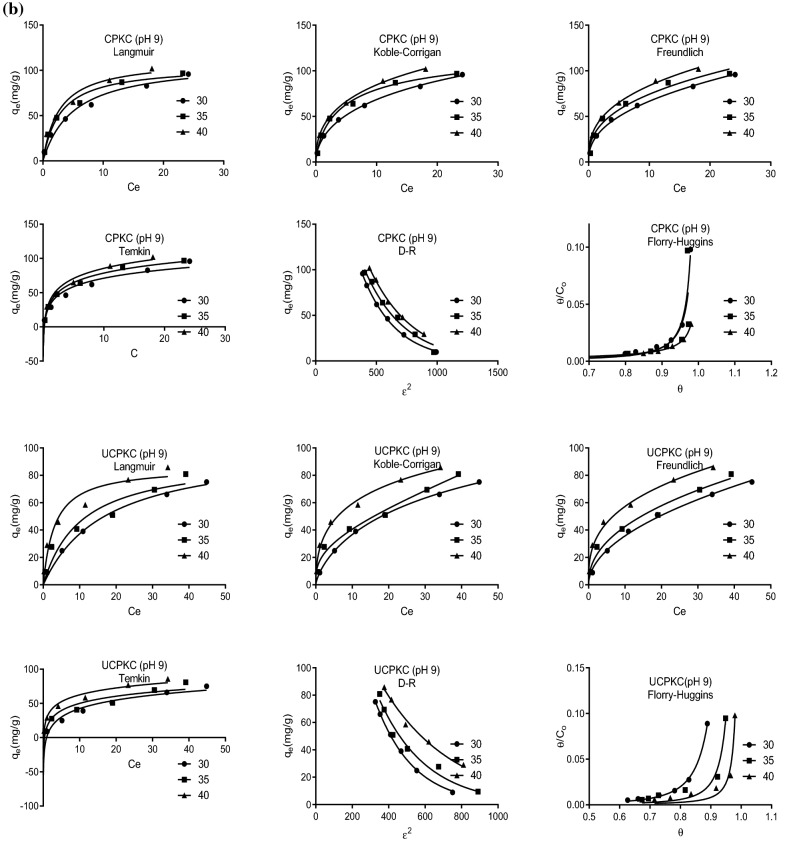
(**a**) Plots of fits of equilibrium data to isotherms at pH 3 and different temperatures. (**b**) Plots of fits of equilibrium data to isotherms at pH 9 and different temperatures.

Consequently, Freundlich isotherm yields a better fit of experimental data at low adsorbate concentration while Langmuir isotherm yields a better fit at high adsorbate concentration^58^. Owing to the foregoing, both linear and nonlinear forms of the isotherms were explored in this study. The nonlinear isotherms used were Langmuir, Freundlich Koble-Corrigan, Temkin, Dubinin-Radushkevich (D-R), and Florry-Huggins isotherms. The linearized isotherms used were Langmuir types I II III and IV, Freundlich, Temkin, D-R, Florry-Huggins, and the generalized isotherm. Figures 3 is the best-fit plots of experimental equilibrium data to selected isotherms. The Koble-Corrigan three-parameter isotherm and the D-R isotherm performed better than all the other isotherms with R^2^ values ranging from 0.97 to 1.0 and 0.95 to 1.0, respectively (Tables 1, 2 and 3). They were closely followed by Freundlich isotherm with an R^2^ value of 0.96 to 1.0 and 0.92 to 1.0 for the nonlinear and linear forms, respectively. The nonlinear form of Temkin isotherm was the least performing of all isotherms investigated with R^2^ values ranging from 0.92 to 0.99 for both linear and nonlinear forms. This can be attributed to the assumption by Temkin isotherm that adsorption involves a uniform distribution of maximum binding energy^57^. This assumption does not apply to PKC being a heterogeneous substance. This might also be responsible for the relatively lower performance of the Langmuir isotherm compared to the other isotherms (0.92 ≤ R^2^ ≤ 1.0). Langmuir isotherm assumes that all binding sites are energetically equivalent. This implies that all active sites have the same probability of binding to the solute upon exposure. Again, while this may be true for homogeneous synthetic materials, it cannot apply to palm kernel chaff which is not homogenous.

**Table 3:** Thermodynamic parameters computed according to Zhou and Zhou^68^ and Milonjic^67^.

T	CPKC (pH 9)			UPKC (pH 9)			CPKC (pH 3)			UPKC (pH 3)		
	∆G (kJ/mol)	∆S (J/mol/K)	∆H (kJ/mol)	∆G (kJ/mol)	∆S (J/mol/K)	∆H (kJ/mol)	∆G (kJ/mol)	∆S (J/mol/K)	∆H (kJ/mol)	∆G (kJ/mol)	∆S (J/mol/K)	∆H (kJ/mol)
**After Milonjic**^64^												
30	−30.7	265.9	−49.7	−27.6	530.8	−133.5	−23.4	189.3	−33.8	−24.1	20.8	17.4
35	−32.7			−29.4			−24.9			−23.0		
40	−33.4			−33.0			−25.2			−24.4		

T	CPKC (pH 9)			UPKC (pH 9)			CPKC (pH 3)			UPKC (pH 3)		
	∆G (kJ/mol)	∆S (J/mol/K)	∆H (kJ/mol)	∆G (kJ/mol)	∆S (J/mol/K)	∆H (kJ/mol)	∆G (kJ/mol)	∆S (J/mol/K)	∆H (kJ/mol)	∆G kJ/mol)	∆S (J/mol/K)	∆H (kJ/mol)
**After Zhou and Zhou**^65^												
30	−33.69	275.72	−49.65	−30.60	540.66	−133.5	−26.33	199.07	−33.81	−27.12	30.58	17.44
35	−35.69			−32.41			−27.89			−26.03		
40	−36.43			−36.03			−28.30			−27.45		

The fact that the Koble-Corrigan three-parameter isotherm integrates both the Langmuir and Freundlich isotherms gave it an edge over other isotherms, particularly the Langmuir and Freundlich isotherms, in fitting the experimental data. The Koble-Corrigan isotherm will reduce to either the Langmuir isotherm or the Freundlich isotherm, depending on experimental conditions. The Koble-Corrigan parameter *n* is equivalent to the inverse of the Freundlich intensity parameter (*1/n*), whereas the isotherm constant *b* is equivalent to Langmuir constant *K*_*L*_. The values of the Koble-Corrigan parameter *n* ranged from 0.45 to 0.73 for CPKC at pH 9, 0.27 to 0.73 for UPKC at pH 9, 0.71 to 1.10 for CPKC at pH 3, and 0.71 to 1.01 for UPKC at pH 3. The corresponding values for Freundlich parameter (*1/n*) are 0.35 to 0.42, 0.34 to 0.51, 0.64 to 0.68 and 0.61 to 0.74. The lowest percentage differences of 3.5% and 4.3% between the two isotherm parameters were calculated for CPKC at 30 °C, pH 9, and UPKC at 35 °C, pH 3. On the other hand, the values of Langmuir constant *K*_*L*_ ranged from 0.197 to 0.369 for CPKC at pH 9, 0.058 to 0.316 for UPKC at pH 9, 0.011 to 0.017 for CPKC at pH 3, and 0.008 to 0.015 for UPKC at pH 3. These values are of the same order of magnitude with the Koble-Corrigan constant (*b*) with corresponding values of 0.106 to 0.29, − 0.203 to 0.151, 0.003 to 0.015, − 0.004 to 0.014. The two parameters have identical values of 0.012 for UPKC at pH 3 and a temperature of 40 °C. However, despite the striking agreement for the case mentioned above, there are wide disparities in a number of cases such as for CPKC at 40 °C and pH 9, UPKC at 35 °C and pH 9, CPKC at 30 °C and pH 3, UPKC at 35 °C and pH 3 where the percentage differences are 215.4%, 147.8%, 266.7%, and 300% respectively. The foregoing suggests that Ni (II) 's adsorption onto palm kernel chaff tends more towards a Freundlich process than a Langmuir process.

Nonetheless, it is expedient to consider in detail the performance of the Langmuir isotherm because its derivation was based on a more mechanistic process than, for instance, the Freundlich isotherm, and it enables the comparison of different adsorbents by using their maximum adsorption capacities (*q*_*max*_)^57^. The nonlinear and linear forms of Langmuir (type II) performed better in the acidic medium of pH 3 with R^2^ values ranging from 0.97 to 1.0 than in the basic medium of pH 9 (0.92 ≤ R^2^ ≤ 0.99). However, the linear Langmuir isotherm (type I) performed relatively better in the basic medium (0.94 ≤ R^2^ ≤ 0.99) than in the acidic medium (0.92 ≤ R^2^ ≤ 0.96). The effect of linearization was immediately obvious with respect to the Langmuir isotherm. The nonlinear and type I linear isotherms were of the same order of accuracy in the basic medium. However, the performance of the type I linear isotherm deteriorated noticeably at pH 3 (0.92 ≤ R^2^ ≤ 0.96). Surprisingly, the type II linearized Langmuir isotherm performed much better (0.96 ≤ R^2^ ≤ 1.0) than the nonlinear isotherm as well as the other three types. Langmuir isotherm (type IV) was the least performing of all isotherms (linear and nonlinear) investigated with R^2^ value ranging from 0.82 to 0.90 for all conditions except for UPKC at pH 9 and 35 °C where a very low R^2^ value of 0.25 was recorded. The Florry-Huggins isotherms performed relatively well (R^2^ = 0.99) at all conditions except for CPKC at 35 °C and pH 9 (R^2^ = 0.52) as a result of undesirable asymptotic behavior observed at a low adsorbate concentration of 10 mg/L. Overall, Temkin and D-R isotherms were hardly affected by the process of linearization. All isotherms that tend towards undesirable asymptotic behavior seem to perform a little better when linearized. Hence, linearization becomes a better and necessary option under such circumstances.

### Adsorbent performance

The sorption capacity of PKC at different conditions was determined based on Langmuir isotherm type II, which yielded the best fit for the experimental data compared to other Langmuir-type isotherms. Careful examination of the isotherm parameters suggests that the other forms of Langmuir isotherm exaggerated the maximum sorption capacity PKC. This can be misleading and result in the wrong sizing of adsorption systems. The range of maximum sorption capacity obtained using the various forms are 34.62 to 110.2, 48.8 to 108.1, 36.0 to 120.6, 41.5 to 99.1, and 47.8 to 104.1 mg/g for the nonlinear and linear types I, II, III, and IV, respectively. Apart from the experiment carried out using CPKC at 35 °C and PH 9, the maximum sorption capacity obtained using the type II isotherm yielded the least value at all conditions. The Langmuir type II isotherm yielded the most reliable values of maximum sorption capacity because it had the highest R^2^ value and the least error. Based on the foregoing, the optimum condition for Ni (II) adsorption onto PKC corresponds to pH 9 and a temperature of 35 °C. Generally, CPKC had better adsorption capacity with maximum sorption capacity (*q*_*max*_) ranging between 65.4 and 120.6 mg/g than UPKC with *q*_*max*_ ranging from 36.0 to 65.9 mg/g. Besides, the sorption capacity of CPKC was significantly enhanced at a basic pH compared to acidic pH. For instance, the maximum sorption capacity of CPKC at PH 9.0 was 120.6 mg/g, which is just about double that of UCPK at pH 3.0. This value of maximum sorption capacity was higher than the values of 102.2 mg/g, 16.6 mg/g, and 29.41 mg/g obtained for the sorption of Ni (II) by protonated rice bran, orange peel, and powder of Mosambi fruit peelings respectively^54^; but agrees very closely with the 120.5 mg/l recorded by Shah et al.^59^ for the sorption of Ni (II) by tea leaf treated with formaldehyde. The performance of the adsorbents was also investigated using the Freundlich intensity parameter (*n*) obtained for the various conditions investigated. As shown in Tables 1, 2 and 3, the values of *n* ranged from 1.98 to 2.97 for pH 9 and 1.35 to 1.63 for pH 3. The values of *n* > 1.0 indicate that the sorption of Ni (II) onto PKC is favorable and that the percentage adsorbed decreases with increasing concentration. All values of *n* obtained in this study were greater than 1.0 were found to be highly correlated with pH (R = 0.9). Higher values of *n* were recorded for both CPKC and UPKC at pH 9 than at pH 3. Hence, the sorption of Ni (II) onto PKC is more favourable at high pH (9) than at lower pH (3). This can be attributed to the values of the zero-point charge (pH_zpc_) of the material which were found to be 4.6 and 4.1 for UPKC and CPKC respectively. pH_zpc_ is the pH of the adsorbent at which the total surface charge is zero, and the values of pH_zpc_ obtained suggest that acid groups are more dominant than basic groups^60^. It can be seen from the pH_zpc_ values that carbonization further reduced the point of zero charge, thus further strengthening the force of attraction between the adsorbent and Ni (II) ions in the solution. This further suggests an affinity with a positively charged ion such as Ni (II). When the pH of the medium is greater than the pH_zpc_, the PKC will assume a net negative surface charge and hence will have increased affinity for the positively charged Ni (II) ion. On the other hand, when the pH of the medium is less than the pH_zpc_, the surface of the adsorbent will assume a net positive charge, resulting in electrostatic repulsion between PKC and Ni (II) and a subsequent reduction in the sorption on Ni (II) onto PKC. This accounts for the reduced performance of PKC at pH 3. It has been established that the sorption of cations will be more favorable at a pH above the pH_zpc_ of the adsorbent, while the adsorption of anions will be more favorable at a pH less than the pH_zpc_ and vice versa^61^. Another reason for the increased performance of the adsorbent at pH 9 is that increase in pH enhances deprotonation of the functional groups on the surface of the palm kernel chaff, thereby creating more negatively charged sites and increasing the performance of the adsorbent^62^. Ghasemi et al.^63^ also reported that as the pH of the solution increased, the sorption of Ni (II) by grape shell ash became more favorable due to the increased negative surface charge of the adsorbent leading to the greater electrostatic attraction between solute and adsorbate. To further explore the favorability of Ni (II) sorption onto PKC, the Langmuir separation factor *R*_*L*_ was determined for all cases studied. The Langmuir separation coefficient is expressed as

18 $${R}_{L}=\frac{1}{1+{K}_{l}{C}_{O}}$$

The value of *R*_*L*_ gives an indication of the relative affinity between adsorbate (Ni) and adsorbent PKC) and is usually classified as follows.

*R*_*L*_ > 1 → unfavourable.

*R*_*L*_ = 1 → linear.

*0* ≤ *R*_*L*_ ≤ 1 → favourable.

*R*_*L*_ = 0 → irreversible.

**Figure 4:**
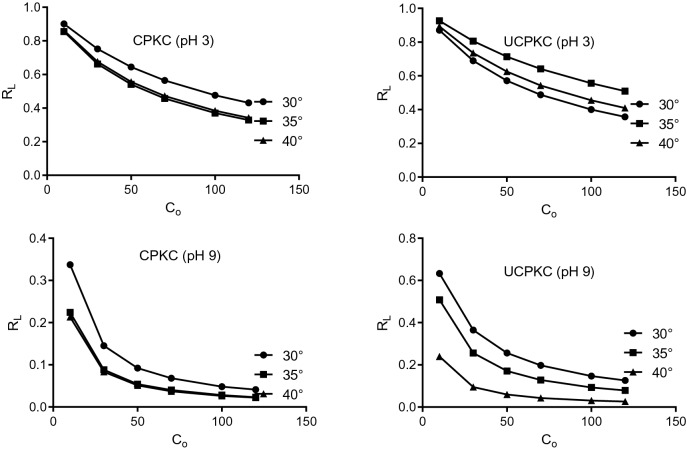
Plots of Langmuir separation factor for different temperatures and pH.

The value of *R*_*L*_ ranged from 0.022 to 0.337, 0.026 to 0.633, 0.342 to 0.901 and 0.41 to 0.926 for CPKC at pH 9, UPKC at pH 9, CPKC at pH 3 and UPKC at pH 3 responsively. This shows that the sorption of Ni (II) onto PKC is very favorable and that there is a strong affinity between PKC and Ni (II). The lowest values of *R*_*L*_ were obtained for CPKC at pH 9 and 40 °C (0.022 ≤ *R*_*L*_ ≤ 0.213), while the highest values were obtained for UPKC at pH 3 and 35 °C (0.51 ≤ *R*_*L*_ ≤ 0.926). The lower the value of *R*_*L*_, the more favorable the sorption process. Figure 4 shows that the sorption of Ni (II) onto PKC is more favorable at higher concentrations of the adsorbate, and this can be attributed to the increased contact between adsorbent and adsorbate as well as increased concentration potential between the solid and liquid phase. The value of *R*_*L*_ tends to 1 at low pH and low concentration, indicating a tendency towards unfavorable conditions.

The sorption mechanism was studied using the activity coefficient (β) of the D-R isotherm. Βeta (β) is related to the sorption energy (*E*) by the expression.

19 $$E= \frac{1}{\sqrt{-2\beta }}$$

**Figure 5:**
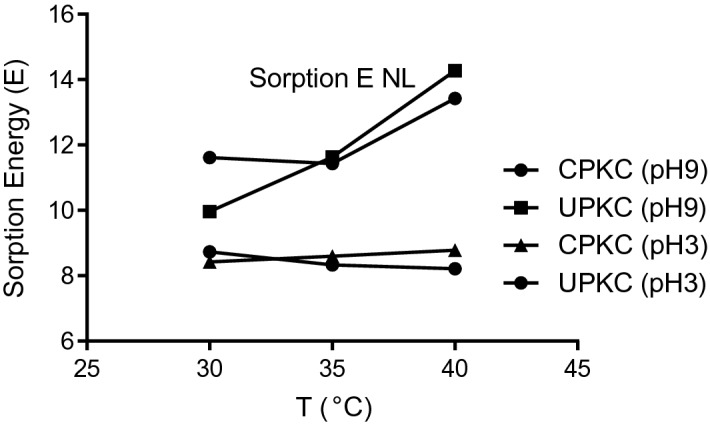
Sorption energy of CPKC and UPKC for different pH.

The sorption energy (*E*) represents the energy required for moving one mole of the solute from infinity to the surface of the adsorbent^57^. Values of *E* < 8 kJ/mol indicate physical sorption; *E* > 16 kJ/mol indicates chemical sorption, while intermediate values of 8 ≤ *E* ≤ 16 kJ/mol signify ion exchange. The values of *E* obtained ranged between 14.27 and 8.21 kJ/mol. These values suggest that the mode of sorption is ion exchange. Shen et al.^62^ identified cation exchange as the predominant mode of sorption of Ni (II) by biochars made from wheat straw pellets and rice husk. The values of *E* ranged from 8.21 to 8.76 kJ/mol at pH 3 and from 9.96 to 14.27 at pH 9. The value of *E* increased with temperature at pH 9 but decreased slightly with the temperature at pH 3. The results reveal that the mode of sorption of Ni (II) onto PKC could be physisorption, ion exchange, or chemisorption, depending on physicochemical conditions under which sorption occurs. Tran et al.^64^ noted that adsorption could be by physisorption, chemisorption, or a mixture of both. Shen et al.^62^ further noted that the sorption of Ni (II) onto biochars was accomplished by other mechanisms besides cation exchange. Figure 5 suggests that the mode of sorption is temperature-dependent and that at higher temperatures than those studied, the sorption process may transit to chemisorption. On the other hand, the process tends towards physisorption at lower pH. Kumar^65^ observed that the forces involved in physical sorption are weak and that the energy requirement is small; hence equilibrium is easily attained and reversible.

The surface coverage of PKC by Ni (II) ions was calculated as follows.

20 $$C= \frac{{q}_{e}}{{q}_{max}}x100$$

**Figure 6:**
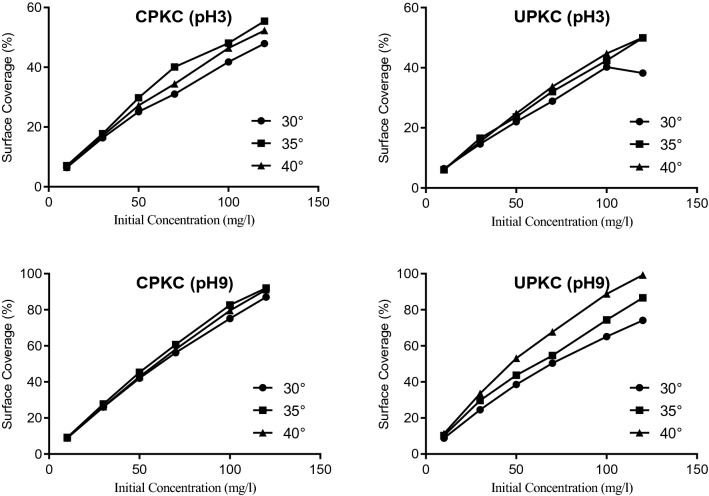
Plots of surface coverage of adsorbents for different temperatures and pH.

Better coverage was obtained at higher temperatures and pH. CPKC recorded higher surface coverage than UPKC (Fig. 6). The maximum coverage obtained for each of the experimental cases was 99.3% for UPKC at pH 9 and 40 °C, 92% for CPKC at pH 9 and 35 °C, 55.4% for CPKC at pH 3 and 35 °C; and 50% for UPKC at pH 3 and 40 °C. Higher values of surface coverage were obtained at higher concentrations of Ni (II) and higher pH. The surface coverage is a measure of the degree of active site utilization by the solute. Hence, higher surface coverage indicates greater utilization of available sites.

### Thermodynamics of Ni (II) adsorption by PKC

Thermodynamics provides a practical tool for estimating the chemical state of adsorbents and the solution in chemical industry application^66^. In order to ascertain the feasibility of the process, Gibbs energy was computed for all experimental conditions. Gibbs energy ∆G° was computed as follows.

21 $$\Delta G^\circ = -RTLn{K}_{o}$$

22 $$\Delta G^\circ =\Delta H -T\Delta S$$

Hence

23 $${LnK}_{0}=\frac{\Delta S}{R} -\frac{\Delta H}{R}\left(\frac{1}{T}\right)$$

where ∆G° is Gibbs energy (kJ/mol), *R* is the universal gas constant (kJ/mol/K), *T* is the temperature (K), ∆H is enthalpy change, and *K*_*o*_ is the standard equilibrium constant. The values of *K*_*o*_ used in this work were obtained using Langmuir constant *K*_*L*_. The major problem with the use of *K*_*L*_ obtained from Langmuir isotherm is that while *K*_*o*_ is dimensionless, *K*_*L*_ has a unit of L/mg. This is a major issue that is often overlooked in adsorption studies. In order to satisfy the requirement that *K*_*o*_ is dimensionless, the values of *K*_*L*_ were modified as suggested by Milonjic^67^ and Zhou and Zhou^68^. While Milonjic^67^ suggested that *K*_*L*_ can be made dimensionless by multiplying with 10^6^, Zhou and Zhou^68^ suggested multiplying by 5500. Applying the above recommendations, Table 3 was generated. ∆S and ∆H were obtained from the intercept and slope of the plot of *Ln K*_*o*_ versus 1/*T*, which yielded a straight-line graph. The values of ∆G° obtained as recommended by Zhou and Zhou^68^ were slightly higher than those obtained by adopting the suggestion of Milonjic^67^. However, the two methods lead to similar deductions with respect to thermodynamic parameters and sorption mechanism. The values of ∆G° obtained were as follows − 33.69 ≤ ∆G° ≤  − 36.43 kJ/mol, − 30.6 ≤ ∆G° ≤  − 36.03, − 26.33 ≤ ∆G° ≤  − 28.3 kJ/mol and − 26.03 ≤ ∆G° ≤  − 27.4 kJ/mol. ∆G° is a measure of the spontaneity of the sorption process, and the negative values suggest that the sorption of Ni (II) onto PKC is feasible and spontaneous. Table 3 shows that the adsorption process is more feasible at pH 9 than at pH 3.

**Figure 7:**
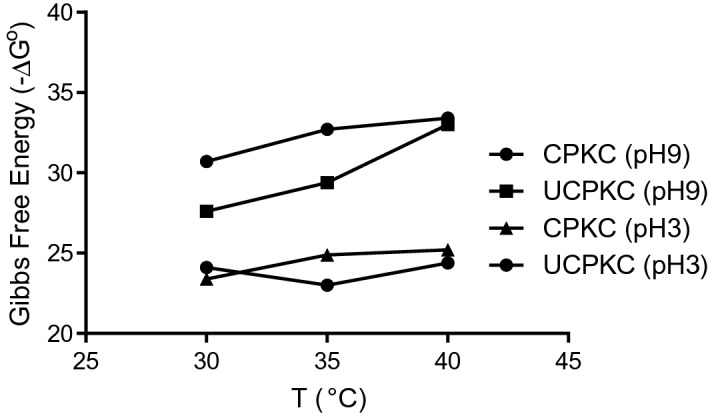
Plots of Gibbs free energy of adsorbents for different pH.

Figure 7 clearly shows that carbonization makes the sorption of Ni (II) onto PKC more energetically feasible by enhancing the porosity of the adsorbent. The values of ∆H obtained are − 49.7, − 133.5, − 33.8, and 17.4 kJ/mol for CPKC at pH 9, UPKC at pH 9, CPKC at pH 3, and UPKC at pH 3, respectively. These values indicate that the process is exothermic for CPKC regardless of pH but exothermic for UPKC at pH 9 and endothermic at pH 3. Shah et al.^59^ also reported that the sorption of Ni (II) by formaldehyde-treated tea leaf was both exothermic and spontaneous. However, Alandis et al.^69^ found that the sorption of Ni (II) by natural bentonite was endothermic. In an exothermic reaction, less energy is required for bond breaking than that required for bond formation. The positive values of ∆S suggest that some structural changes occurred on the adsorbent and confirmed that affinity exists between the adsorbent and adsorbate^58^. The values of ∆S° also suggest that the process is entropy-driven. Saha and Chowdhury^21^ observed that the heat evolved during physical sorption is of the same order of magnitude as the heat of condensation (2.1–20.9 kJ/mol), while the heat of chemisorption generally falls within the values of 80–200 kJ/mol^66^. The values of enthalpy change (17.44 ≤ ∆H° ≤ 49.65 kJ/mol) obtained in this study lies between these two ranges aforementioned for all cases investigated except for UPKC at pH 9 with ∆H° = 133.5 kJ/mol. This suggests that the sorption of Ni (II) onto PKC can be accomplished either by chemisorption or ion exchange, depending on experimental conditions. The values of ∆H° for UPKC at pH 9 suggest chemisorption, but the corresponding values of sorption energy (8.21 ≤ E ≤ 14.27) suggest otherwise. In order to further understand the adsorption mechanism, the isosteric heat of adsorption (∆H_x_) was computed. According to Saha and Chowdhury^21^, the isosteric heat of sorption is the most relevant thermodynamic property for describing heat effects during adsorption process. It is a very important parameter for characterization and optimization of adsorption process which also gives an indication about the surface heterogeneity of the adsorbent^21^. The expression for the isosteric heat of sorption is given below.

24 $$Ln{C}_{e}= -\left[\frac{\Delta {H}_{x}}{R}\right]\frac{1}{T}+K$$

*K* is a constant while *C*_*e*_ is the equilibrium concentration of Ni (II) in solution and the other parameters retain their usual meanings as earlier defined. The isosteric heat of sorption ∆H_x_ was obtained from the slope of plots of *Ln C*_*e*_ versus *1/T*. The values of ∆H_x_ obtained were − 62.0 ≤ ∆H_x_ ≤  − 22.68, − 133.09 ≤ ∆H_x_ ≤  − 21.34, − 33.87 ≤ ∆H_x_ ≤  − 17.92 and − 24 ≤ ∆H_x_ ≤  − 19.21 kJ/mol for CPKC at pH 9, UPKC at pH 9, CPKC at pH 3 and UPKC at pH 3. Twenty-two (22) of the 24 values of ∆H_x_ were less than 80 kJ/mol, while 23 of the 24 values were greater than 20.9 kJ/mol suggesting an intermediate state between physisorption and chemisorption. Saha and Chowdhury^21^ noted that for chemical sorption, ∆H_x_ should range between 80 and 400 kJ/mol.

**Figure 8:**
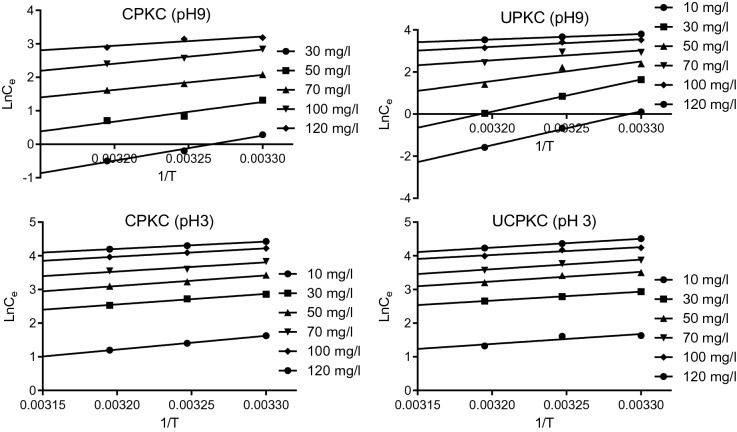
Plots of *LnC*_*e*_ versus *1/T* for determination of isosteric heat of sorption.

Contrary to the findings of this study, Temel^70^ reported that the sorption of Ni (II) by *gyttja* was purely by physical sorption. Physisorption usually involves weak Van der Waal forces, ion exchange involves coulombic forces. In contrast, chemisorption involves the formation of a covalent bond between the solute and the surface of the adsorbent. The variation of ∆H_x_ with surface loading (Fig. 8) indicates that the adsorbents have energetically heterogeneous surfaces. Higher values of ∆H_x_ were obtained at low adsorbate concentration suggesting the existence of highly active sites on the surfaces of the adsorbents^21^.

### Kinetics of Ni (II) adsorption by PKC

A Kinetic study was designed as an integral part of the experiment process. The agitation period was set at 5, 10, 20, 40, 60, 90, and 120 min. The first-order kinetic, second-order kinetic, pseudo-first-order kinetic, pseudo-second-order kinetic, and the inter-particle diffusion models were applied to model the sorption rate Ni(II). The first-order kinetic model and its integrated form are given respectively in Eqs. 25 and 26.

25 $$\frac{dC}{dt}= -KC$$

26 $${L}_{n}\frac{{C}_{o}}{C}=Kt$$

The second-order kinetic model and its integrated form are given respectively in Eq. 3.9 and Eq. 3.10,

27 $$\frac{dC}{dt}= {-KC}^{2}$$

28 $$\frac{1}{C}= \frac{1}{{C}_{o}}+Kt$$

where $C$ is the solute concentration in solution $(mg/L)$ at time $t (mins)$ and $K$ is the rate constant. The pseudo-first-order kinetic model is as follows:

29 $$\frac{{dq}_{t}}{dt}=K\left({q}_{e}- {q}_{t}\right)$$

where ${q}_{t}$ represents the amount of solute adsorbed per gram of the adsorbent $(mg/g)$ and ${q}_{e}$ is the equilibrium concentration of solute in the adsorbent $(mg/g)$. The term $\left({q}_{e}- {q}_{t}\right)$ represents the sorption driving force. In the same vein, the integrated and linearized form of the pseudo-second-order kinetic model is given in Eq. (30).

30 $$\frac{t}{{q}_{t}}= \frac{1}{{{Kq}_{e}}^{2}}+ \frac{t}{{q}_{e}}$$

The intraparticle diffusion model is given as Eq. (31)

31 $${q}_{t}= {K}_{p} \sqrt{t}+ {I}_{d}$$

where ${K}_{p}$ is the initial rate of intraparticle diffusion and ${I}_{d}$ is the model constant.

A summary of the coefficient of correlation *R*^2^ and the rate constant *k* is presented in Tables 4, 5, 6 and 7. At the temperatures (30 °C, 35 °C, and 40 °C), pH (3 & 9), initial metal concentrations (10, 30, 50, 70, 100 & 120 mg/l) and adsorbent types (UCPKC & CPKC) studied, the pseudo-second-order kinetic model shows best correlation compared to the other kinetic models. The coefficients of correlation for the pseudo-second-order model were mostly within the range of 0.9990–1.0000 for 90% of all kinetic studies carried out, as shown in Tables 4, 5, 6 and 7. The best rate-determining model of biosorption for this study was Ho’s pseudo-second-order kinetic model, followed closely by the intra-particle diffusion model. The best coefficients of correlation were obtained at pH 9 for CPKC and UCPKC. Generally, R^2^ ≥ 0.99 was obtained for more than 90% of the results of the kinetic study. At high solute concentrations, the availability of sorption sites in the adsorbent is a critical factor in determining the rate of biosorption, hence the performance of the pseudo-second-order kinetic model. The pseudo-second-order model assumes that the biosorption of solutes follows second-order chemisorption. At low solute concentrations, however, the first order and second-order kinetic models perform better^37,43^. The worst performance of the pseudo-second-order kinetic model occurred at UCPKC at pH 3 with average R^2^ ≤ 0.95. This meant that there were less readily available biosorption sites for the solutes at all temperature conditions studied for UCPKC at pH 3. Gonen et al.^71^ also reported that pseudo-second-order and intraparticle diffusion kinetic models were the best rate-determining models with R^2^ > 0.99 for the biosorption of Ni(II) using orange peel. In addition, Arshadi et al.^54^ (2014), in the study of the adsorption of Ni(II), Cd(II), Cu(II), and Co(II) ions onto barley straw ash, confirmed that the rate-limiting model was pseudo-second-order kinetic. This increases the likelihood that the biosorption of Ni (II) using Palm Kernel Chaff is a chemisorption process.

**Table 4:** R^2^ and K values for various kinetic models for CPKC at pH 9 and Temperatures 30 °C, 35 °C and 40 °C.

Ads	pH	Temp (°C)	Initial Conc. (mg/l)	First order		Pseudo first order		Second order		Pseudo second order		Intra particle diffusion	
				k (min^−1^)	R^2^	k (min^−1^)	R^2^	k (l/mg min)	R^2^	k (g/mg min)	R^2^	k (g/mg min^−1^)	R^2^
CPKC	9	30	10	3.0351	0.5011	−0.0883	0.9249	2.3267	0.5514	0.0256	1.0000	9.2667	0.6276
			30	2.3599	0.9593	1.9156	0.8277	0.3302	0.9591	0.0250	0.9999	26.7440	0.9625
			50	2.1967	0.9688	1.7641	0.9439	0.1776	0.9855	0.0114	0.9999	43.8338	0.9891
			70	1.8698	0.6874	2.7901	0.6547	0.0928	0.7219	0.0078	1.0000	57.7591	0.8012
			100	1.6870	0.9218	1.2965	0.9175	0.0540	0.9259	0.0025	1.0000	80.9729	0.9795
			120	1.5338	0.9092	1.7365	0.9026	0.0386	0.9157	0.0025	1.0000	93.4240	0.9801
		35	10	3.1510	0.7355	−1.4054	0.7355	2.3184	0.7379	0.0184	1.0000	9.5256	0.7511
			30	2.7144	0.9523	0.3557	0.9680	0.4630	0.9815	0.0172	1.0000	27.6529	0.9607
			50	2.6684	0.7446	1.9503	0.7005	0.2875	0.7580	0.0057	1.0000	45.9437	0.8618
			70	2.2086	0.9392	0.7820	0.9470	0.1295	0.9509	0.0052	1.0000	61.7360	0.9682
			100	1.9195	0.8572	0.8111	0.9937	0.0681	0.8675	0.0026	1.0000	84.6580	0.9475
			120	1.5920	0.8225	0.5161	0.8932	0.0409	0.8260	0.0016	1.0000	95.0445	0.9195
		40	10	3.8379	0.6676	−1.5353	0.6676	4.6430	0.6711	0.0007	1.0000	15.5750	0.6748
			30	2.9316	0.9621	0.1126	0.9622	0.5623	0.9818	0.0141	1.0000	28.1036	0.9714
			50	2.7963	0.8438	0.8640	0.9255	0.3248	0.8719	0.0054	1.0000	46.4974	0.9196
			70	2.3669	0.7764	1.6006	0.9361	0.1521	0.7902	0.0043	1.0000	62.6882	0.8843
			100	2.0789	0.8659	0.6894	0.9822	0.0799	0.8771	0.0023	1.0000	86.8727	0.9536
			120	1.8312	0.7939	0.5256	0.8918	0.0520	0.7981	0.0013	1.0000	100.2499	0.9059

**Table 5:** R^2^ and K values for various kinetic models for UPKC at pH 9 and Temperatures 30 °C, 35 °C and 40 °C.

Ads	pH	Temp (°C)	Initial Conc. (mg/l)	First order		Pseudo first order		Second order		Pseudo second order		Intra particle diffusion	
				k (min^−1^)	R^2^	k (min^−1^)	R^2^	k (l/mg min)	R^2^	k (g/mg min)	R^2^	k (g/mg min^−1^)	R^2^
UCPKC	9	30	10	1.5732	0.9511	0.2672	0.9855	0.4655	0.9792	0.1642	0.9997	7.5911	0.9820
			30	1.4874	0.9734	0.4802	0.9824	0.1465	0.9660	0.0383	0.9995	22.7718	0.9507
			50	1.4055	0.9644	0.3771	0.9786	0.0815	0.9697	0.0120	0.9999	37.2910	0.9846
			70	1.1933	0.9406	1.2175	0.7824	0.0471	0.9399	0.0136	0.9998	48.0076	0.9185
			100	1.0336	0.9623	0.6833	0.9971	0.0281	0.9651	0.0052	1.0000	63.8299	0.9979
			120	0.9658	0.7601	−0.0017	0.8377	0.0219	0.7622	0.0017	1.0000	73.9446	0.8746
		35	10	2.2469	0.5641	0.3660	0.8305	0.9992	0.6167	0.0618	0.9999	8.5338	0.6779
			30	1.8362	0.7894	1.2928	0.9627	0.2047	0.8834	0.0376	0.9999	24.0062	0.8141
			50	1.5159	0.9369	0.7286	0.8393	0.0908	0.9404	0.0162	0.9998	38.4883	0.8939
			70	1.2351	0.9648	0.3126	0.9512	0.0491	0.9661	0.0071	0.9999	49.2300	0.9600
			100	1.1344	0.9412	0.4879	0.8711	0.0311	0.9407	0.0049	0.9999	67.3559	0.9084
			120	1.0831	0.9466	0.8645	0.8410	0.0246	0.9471	0.0033	1.0000	78.8357	0.9510
		40	10	2.9231	0.7833	−0.8055	0.8060	1.7519	0.8750	0.0392	1.0000	9.2972	0.7627
			30	2.5373	0.8911	0.4433	0.9341	0.3924	0.9057	0.0200	0.9999	27.1481	0.9257
			50	2.3951	0.7114	−0.6053	0.7718	0.2194	0.7235	0.0024	1.0000	45.2235	0.8404
			70	1.6345	0.9589	0.9591	0.9916	0.0731	0.9681	0.0085	0.9999	55.5791	0.9947
			100	1.3903	0.8435	0.7163	0.9715	0.0402	0.8504	0.0033	1.0000	74.4177	0.9414
			120	1.2373	0.6618	−0.2692	0.8962	0.0287	0.6658	0.0010	1.0000	84.8953	0.7816

**Table 6:** R^2^ and K values for various kinetic models for CPKC at pH 3 and Temperatures 30 °C, 35 °C and 40 °C.

Ads	pH	Temp (°C)	Initial Conc. (mg/l)	First order		Pseudo first order		Second order		Pseudo second order		Intra particle diffusion	
				k (min^−1^)	R^2^	k (min^−1^)	R^2^	k (l/mg min)	R^2^	k (g/mg min)	R^2^	k (g/mg min^−1^)	R^2^
CPKC	3	30	10	0.1605	0.9165	1.5268	0.9724	0.1150	0.9464	4.0891	0.9507	0.2383	0.9486
			30	0.2827	0.8983	1.9841	0.9120	0.0440	0.9069	0.6410	0.9875	5.4980	0.9270
			50	0.2548	0.9603	2.2943	0.9936	0.0257	0.9725	0.4102	0.9919	8.3930	0.9962
			70	0.2591	0.8636	2.4968	0.9764	0.0185	0.8781	0.2232	0.9978	12.6558	0.9533
			100	0.3219	0.9266	1.9023	0.7938	0.0138	0.9274	0.0682	0.9985	25.9924	0.9291
			120	0.3124	0.9145	2.0097	0.8875	0.0114	0.9171	0.0468	0.9995	30.4748	0.9717
		35	10	0.2001	0.9585	1.7399	0.9483	0.1161	0.9763	3.1696	0.9561	0.5246	0.9686
			30	0.4845	0.9148	1.5606	0.9726	0.0540	0.9267	0.2296	0.9985	10.2379	0.9723
			50	0.4305	0.9103	2.1355	0.9978	0.0306	0.9145	0.2005	0.9949	14.7029	0.9578
			70	0.4955	0.8867	2.0315	0.9464	0.0234	0.9004	0.0799	0.9988	24.9998	0.9454
			100	0.4410	0.7166	1.5937	0.7949	0.0155	0.7251	0.0330	0.9997	33.6861	0.8377
			120	0.4243	0.9446	1.7959	0.9464	0.0127	0.9469	0.0321	0.9995	39.8299	0.9647
		40	10	0.2977	0.9420	1.7954	0.9483	0.1255	0.9655	2.1567	0.9720	1.2820	0.9533
			30	0.5665	0.9532	1.8944	0.8387	0.0582	0.9539	0.2535	0.9950	11.5232	0.9507
			50	0.5586	0.8174	2.2815	0.9825	0.0349	0.8457	0.1172	0.9994	18.4720	0.9134
			70	0.4865	0.8860	2.7040	0.8869	0.0232	0.9019	0.1018	0.9980	23.6001	0.9538
			100	0.4398	0.9268	2.7977	0.9848	0.0155	0.9391	0.0835	0.9980	31.0178	0.9787
			120	0.4430	0.8442	2.4824	0.8520	0.0130	0.8576	0.0523	0.9984	38.7346	0.9238

**Table 7:** R^2^ and K values for various kinetic models for UPKC at pH 3 and Temperatures 30 °C, 35 °C and 40 °C.

Ads	pH	Temp (°C)	Initial Conc. (mg/l)	First order		Pseudo first order		Second order		Pseudo second order		Intra particle diffusion	
				k (min^−1^)	R^2^	k (min^−1^)	R^2^	k (l/mg min)	R^2^	k (g/mg min)	R^2^	k (g/mg min^−1^)	R^2^
UCPKC	3	30	10	0.0761	0.9198	1.4652	0.8627	0.1047	0.9397	9.7948	0.4123	−0.5892	0.9384
			30	0.2378	0.9195	1.5084	0.8909	0.0421	0.9072	0.8288	0.9555	5.1751	0.8562
			50	0.1671	0.9320	2.6435	0.9164	0.0235	0.9418	0.7315	0.9786	4.3126	0.9683
			70	0.2506	0.5009	1.8936	0.6490	0.0184	0.5286	0.2014	0.9968	12.1198	0.6033
			100	0.3113	0.9382	1.6020	0.9015	0.0136	0.9404	0.0658	0.9989	25.4036	0.9355
			120	0.1662	0.4526	4.6450	0.7549	0.0099	0.4761	0.2504	0.9871	12.2134	0.5614
		35	10	0.2036	0.9307	3.3036	0.7414	0.1202	0.9477	3.1922	0.9665	0.7739	0.9555
			30	0.4320	0.5289	1.2575	0.7236	0.0515	0.5659	0.2172	0.9990	8.7344	0.6397
			50	0.2469	0.7483	2.5590	0.9799	0.0256	0.7551	0.4160	0.9904	6.6615	0.8627
			70	0.3518	0.8094	1.8684	0.8283	0.0203	0.8289	0.1272	0.9968	18.4895	0.8651
			100	0.3737	0.9284	1.8098	0.8498	0.0145	0.9306	0.0500	0.9992	29.7023	0.9475
			120	0.3344	0.8725	4.4502	0.7619	0.0116	0.8805	0.0619	0.9993	30.9806	0.9563
		40	10	0.2653	0.9470	3.5678	0.6810	0.1234	0.9240	2.4143	0.9412	1.3579	0.9479
			30	0.5332	0.8652	3.4724	0.7078	0.0567	0.8770	0.2089	0.9969	11.2197	0.8931
			50	0.2423	0.9104	3.5228	0.8265	0.0250	0.9216	0.5162	0.9795	5.3090	0.9578
			70	0.3787	0.5623	2.5603	0.6716	0.0210	0.6294	0.1813	0.9935	15.4148	0.6344
			100	0.5578	0.7210	1.3261	0.9457	0.0175	0.7310	0.0167	0.9999	41.3680	0.8469
			120	0.4517	0.6652	2.0127	0.6629	0.0131	0.6868	0.0391	0.9985	40.6005	0.7361

## Conclusion

PKC makes an excellent biosorbent at pH 9.0 and 40 °C, especially when carbonized before chemical activation. Hence, CPKC generally had a higher adsorption capacity than UPKC. Complete removal (100%) of Ni (II) from solution was recorded for CPKC at pH 9 in 60 min. One gram (1 g) of the biosorbent can remove as much as 120.6 mg of nickel from an aqueous solution by mostly ion exchange for a complete monolayer coverage. This was higher than the maximum monolayer adsorption capacity recorded by other researchers for rice bran, orange peel, and powder of Mosambi fruit peelings. The sorption process was more favorable in the basic medium (pH 9) than in the acidic medium (pH 3) because the point of zero charge which was 4.6 for UPKC and 4.1 for CPKC. The Koble-Corrigan and D-R isotherms adequately described the sorption of Ni (II) by palm kernel chaff with very high coefficients of correlation. The Freundlich isotherm was also found to fit the experimental data. The linear type II Langmuir isotherm performed much better than both the nonlinear type as well as the other linear types. The values of Freundlich intensity parameters (*n*) and Langmuir separation coefficients (*R*_*L*_) revealed that the sorption on Ni (II) onto PKC is more favorable at higher solute concentrations. The values of sorption energy (*E*) and isosteric heat of sorption (∆H_x_) confirmed that the sorption process is an ion exchange process under the physicochemical conditions used in the study. The sorption of Ni (II) onto PKC is exothermic, spontaneous, entropy-driven, and highly feasible. Higher values of isosteric heat of sorption (∆H_x_) at low adsorbate concentrations suggest the availability of highly active sorption sites on the surface of the adsorbents.
